# O9-4 Barriers and facilitators in Belgian inactive desktop workers to participate in workplace based physical activity programs

**DOI:** 10.1093/eurpub/ckac094.068

**Published:** 2022-08-29

**Authors:** Ine De Clerck, Dries Van Hulle, Annelies Maenhout

**Affiliations:** 1 Health & Care, Artevelde University of Applied Sciences, Gent, Belgium; 2 Physical activity & Physical Recreation, Artevelde University of Applied Sciences, Ghent, Belgium; 3 Occupational Therapy, Artevelde University of Applied Sciences, Ghent, Belgium

**Keywords:** Workplace based physical activity, inactive employees, barriers, facilitators

## Abstract

**Background:**

Workplace based physical activity programs show great potential to improve health and wellbeing to a large part of the population. However, these programs appear to recruit mostly participants who already have an active lifestyle. This study aims to identify barriers and facilitators of inactive desktop workers, and can serve to inform policy makers or physical activity program designers to adapt current program in order to reach more inactive participants.

**Methods:**

Desktop workers were voluntarily recruited by use of social media and by direct communication to companies. Inactive participants were included by means of the International Physical Activity Questionnaire (IPAQ). Semi-structured interviews were conducted with 22 desktop workers, being employed in 15 different Belgian companies and being mostly female (n = 18). Interviews were transcribed verbatim and analysed using thematic analysis.

**Results:**

Results indicate that interpersonal relationships can be both a potential facilitator (e.g. having a colleague who asks you to join the exercise program) as a barrier (e.g. being scared of negative reactions from colleagues). Furthermore, organisational factors such as a high workload or a perceived lack of time is claimed as an important mediating factor for participation. On the other hand, having permission to exercise during working hours or having a fixed time to exercise said to be an important facilitator. Also environmental factors are important, since not having an on-site physical activity facility or program is seen as an important barrier. Lastly, also communication from the management being supportive and emphasizing the importance of participating is found to be an important motivator.

**Conclusions:**

This study shows several specific barriers and facilitators of inactive desktop workers to participate within workplace based physical activity programs. These results can support developers of physical activity programs to create more targeted interventions in order to reach more inactive employees.

